# Triple-Network Functional Connectivity in Adolescents With Autism Spectrum Disorder Compared to Early-Onset Psychosis

**DOI:** 10.1002/aur.70163

**Published:** 2026-03-28

**Authors:** Aarti Nair, Rhideeta Jalal, Katherine E. Lawrence, Niharika Verma, Hector Gutierrez, Kristen Laulette, Katherine H. Karlsgodt, Carrie E. Bearden

**Affiliations:** 1 Department of Psychology, Loma Linda University, Loma Linda, California, USA; 2 Imaging Genetics Center, Mark and Mary Stevens Neuroimaging and Informatics Institute, University of Southern California, Los Angeles, California, USA; 3 Department of Psychology, University of California, Los Angeles, California, USA; 4 Department of Psychiatry and Biobehavioral Sciences, Semel Institute for Neuroscience and Human Behavior, University of California, Los Angeles, California, USA

**Keywords:** autism spectrum disorder, early-onset psychosis, functional connectivity, resting-state, social cognition

## Abstract

Both autism spectrum disorder (ASD) and psychosis are associated with challenges in social cognition. The triple-network model posits that dysfunction within and between the default mode network (DMN), central executive network (CEN), and salience network (SN) contributes to these deficits. However, the relationship between triple-network connectivity and social cognition has not been systematically compared across these groups during adolescence. We examined whole-brain functional connectivity of the triple-network in a sample of youth with ASD (*N* = 24), youth with early-onset psychosis (EOP; *N* = 25), and age-matched typically developing (TD) controls (*N* = 26, overall mean age = 16.39 ± 2.36, % female = 41%). Additionally, we examined relationships between connectivity patterns of the triple-network and behavioral measures of social cognition and emotion recognition in each group. ASD youth showed mixed over- and under-connectivity in the DMN, CEN overconnectivity, and SN underconnectivity, while EOP youth showed DMN and CEN overconnectivity, with relatively intact SN connectivity, compared to TD controls. Compared to EOP, ASD participants had reduced SN connectivity and mixed disruptions in DMN connectivity. Across groups, connectivity patterns were linked to social behaviors: in EOP, DMN overconnectivity was associated with poorer social cognition; in ASD, SN underconnectivity was associated with poorer social cognition. These findings highlight both shared and distinct patterns of triple-network dysconnectivity in ASD and EOP, supporting transdiagnostic models of social cognitive dysfunction, and reinforcing adolescence as a key developmental window for network-based brain changes.

## Lay Summary

Youth with autism spectrum disorder (ASD) and early-onset psychosis (EOP) can both struggle to understand and respond to social situations. In this study, we used resting-state MRI scans to examine how three key brain networks—those involved in theory of mind, attention, and detecting socially salient information—function in adolescents with ASD and EOP compared to neurotypical youth. We found different patterns of brain connectivity in each group, with ASD youth showing weaker connections in the brain network that helps to notice and respond to important social cues, and in contrast, EOP youth showing stronger-than-usual connections in key regions for attention and working memory. Subsequently, these patterns were linked to how well participants performed on tasks involving emotion recognition and social cognition. These findings suggest that social difficulties in ASD and EOP may stem from differing patterns of disruptions in similar social brain networks.

## 1 | Introduction

Difficulties in social cognition are a hallmark feature of several neuropsychiatric disorders, notably autism spectrum disorder (ASD) and early-onset psychosis (EOP; Pinkham et al. 2008). These heterogeneous conditions share several overlapping phenotypic features as well as some common genetic risk factors (Craddock and Owen 2010; Morrison et al. 2017; Jutla et al. 2022; Skuse 2022). ASD is a prevalent neurodevelopmental disorder, characterized by challenges in social reciprocity and communication, as well as the presence of restrictive, repetitive behaviors, and circumscribed interests (American Psychiatric Association 2013). The prevalence of ASD is estimated to affect about 2%–3% of children, and has a typical age onset before age 4 (Chihuri et al. 2024). Psychotic disorders are severe and chronic mental health disorders, characterized by positive symptoms such as hallucinations, delusions, disorganized speech, as well as negative symptoms such as lack of emotional reactivity and social withdrawal (American Psychiatric Association 2013). Psychotic symptoms typically manifest during adolescence and early adulthood, although approximately 11%–18% of patients have onset of their first psychotic episode occurring before age 18, thereby categorizing them as EOP (Díaz-Caneja et al. 2015).

Although ASD and EOP differ in the presentation of symptoms, age of onset, and developmental course, the behavioral overlap between ASD and psychosis is substantially characterized by impaired social functioning (Diagnostic and Statistical Manual of Mental Disorders, 5th ed., text rev.; DSM-V-TR; American Psychiatric Association 2022), which may relate to challenges in social cognition (Pinkham et al. 2003, 2008). Several studies have found that individuals with ASD (Bookheimer et al. 2008; Berenguer et al. 2018; Eack et al. 2013) and psychosis (Mehl et al. 2020; Thompson et al. 2012) exhibit similar social cognition difficulties, such as impairments in understanding social cues, recognizing emotions in others, and interpreting social intentions, as well as deficits in face-emotion perception, affective empathy, and theory of mind (ToM; Bora et al. 2009; Rosenthal et al. 2019). Social cognitive and neurocognitive performance is impacted in both autism and schizophrenia spectrum disorders (SSD), with SSD showing more pronounced neurocognitive deficits (Rashidi et al. 2025). However, the timing of the onset of these social challenges varies; for instance, in ASD, deficits typically manifest very early in development and can be detected as social behaviors begin to develop. In contrast, the manifestation of social deficits in EOP can vary significantly, as some individuals may experience insidious and longstanding deficits, while others may experience a more acute onset of social impairments (De la Serna et al. 2023; Manolesou and Kyriakopoulos 2024).

Adolescence, in particular, is a critical period for social development, in which social demands change most dramatically. The adolescent phase plays a pivotal role in the maturation of brain regions associated with social cognition and self-awareness, which may likely arise from a complex interplay of factors such as alterations in the social milieu, hormonal shifts during puberty, and both structural and functional brain developmental changes, all contributing to gains in social cognition (Blakemore 2008). Previous studies suggest that social changes in the brain during adolescence may include significant maturation in perspective taking (Sebastian et al. 2012) and face processing skills (Kadosh et al. 2013), while also encompassing increases in empathy (Blakemore 2008). Social impairments become increasingly apparent during this phase, as social contexts grow in complexity and present greater social expectations (Happé and Frith 2014). The consequences of social challenges encompass peer rejection or victimization, diminished friendship quality, elevated rates of depression and loneliness, and diminished self-esteem (Bauminger and Kasari 2000; Rao et al. 2008).

There are three interrelated functional brain networks, often referred to as the triple-network model, that may offer a framework for the observed overlap of social impairment symptoms among individuals with ASD or EOP, including differences in social cognition, executive function, and salience attribution (Ruta et al. 2017; Uddin 2015). The triple-network hypothesis (Menon 2011) suggests that many psychiatric and neurological conditions arise from disruptions in the dynamic associations between the default mode network (DMN), central executive network (CEN), and salience network (SN). The SN plays a key role in mediating the balance between the DMN, which is involved in self-referential thinking, and the CEN, responsible for goal-oriented tasks and cognitive control. Dysfunction within or between these networks can impair cognitive flexibility, leading to the social and cognitive deficits seen in ASD and EOP (Menon 2011; Uddin 2015). The DMN is most active during rest and self-focused thought (Menon 2011), while the CEN supports executive functions like decision-making and working memory (Seeley et al. 2007). The SN facilitates switching between networks by detecting and responding to salient environmental cues (Menon and Uddin 2010; Goulden et al. 2014). This triple-network model, as such, provides a framework to examine brain networks supporting differences in cognitive, affective, and social functions that have been implicated in several neuropsychiatric disorders, including ASD and EOP (Liang et al. 2021; Menon 2011; Wang et al. 2020).

Previously, separate studies have examined the triple-network model among individuals with ASD, EOP, and schizophrenia (Krishnadas et al. 2014; Abbott et al. 2016; Menon 2018; Lawrence et al. 2019, 2020; O’Neill et al. 2019; Wang et al. 2020). Only two studies thus far have directly compared DMN connectivity in matched ASD and EOP groups (Ciaramidaro et al. 2015; Hyatt et al. 2020), while no published studies have examined CEN and SN connectivity across these groups.

Given the dearth of studies examining the triple-network collectively in comparative samples of adolescents with ASD and EOP, here we look at alterations in the DMN, CEN, SN whole-brain functional connectivity in these groups, and in typically developing (TD) controls using resting-state functional connectivity MRI (rs-fcMRI). To the best of our knowledge, this study represents the first rs-fcMRI study to investigate the triple-network and its association with social cognition in demographically matched youth with ASD, EOP, and TD controls. Additionally, we examine for the first time how connectivity patterns for each network are associated with behavioral measures of social cognition in ASD and EOP, in order to determine how triple-network connectivity in each group may relate to social perspective taking and emotion recognition, aspects of social functioning impacted in both ASD and EOP. Importantly, understanding the nature of such differences would allow us to better characterize the brain-based underpinnings of social cognition difficulties among these often impairing conditions and shed new light on the potential relationship between brain connectivity differences and behavioral markers of social functioning. Moreover, network alterations in childhood and adolescence have been shown to later impact social communication abilities and functional outcomes (Uddin et al. 2013; Menon 2018; Keehn et al. 2021), and therefore, this work may also provide insight into targets for intervention and potential biomarkers of prognosis. Based on prior literature, we hypothesized that both ASD and EOP youth would exhibit disruptions in triple-network connectivity compared to TD controls, but with distinct profiles. Specifically, we expected ASD youth to exhibit mixed DMN over- and under-connectivity and SN underconnectivity compared to TD controls, and EOP youth to exhibit DMN and CEN overconnectivity, with mixed under- and over-connectivity of the SN compared to TD controls. We further expected ASD youth to exhibit mixed under- and over-connectivity of the DMN, overconnectivity of the CEN, and underconnectivity of the SN compared to EOP youth. We lastly hypothesized that these altered connectivity patterns would relate to social cognition performance, such that mixed DMN connectivity and SN underconnectivity would be associated with poorer social cognition in ASD, and DMN and CEN overconnectivity would be associated with poorer social cognition in EOP.

## 2 | Materials and Methods

### 2.1 | Participants

Participants aged 12–21 years were recruited via voluntary response sampling techniques through posting of recruitment flyers at various University of California, Los Angeles (UCLA) outpatient psychiatric clinics, as well as outreach efforts at various public events in the Southern California region. Individuals with ASD were primarily recruited from ongoing research studies at the UCLA’s Center for Autism Research and Treatment (CART). For the ASD group, all participants were required to have a community diagnosis of ASD, confirmed by an experienced, research-reliable clinician. Participants were assessed for ASD symptomatology using the Autism Diagnostic Observation Schedule, 2nd edition (Lord et al. 2012; see Supporting Information for detailed description of clinical measures) and best clinical judgment. EOP individuals were recruited from the UCLA Adolescent Brain-Behavior Research Center (ABBRC). Inclusion criteria for EOP participants involved a diagnosis of psychotic disorder (including schizophrenia, schizophreniform disorder, schizoaffective disorder, bipolar I disorder or major depressive disorder with psychotic features, and psychotic disorder not otherwise specified), confirmed using the Structured Clinical Interview for DSM-V Axis I diagnoses (SCID-5; First et al. 2015) by trained study personnel. TD controls were recruited from each of these ongoing studies and community outreach efforts. Inclusion criteria for TD controls included no learning disabilities, no personal or close family history of any major developmental, psychiatric or neurological disorders, and the existence of any first-degree relative with ASD or EOP. Exclusionary criteria for all groups included full-scale IQ < 70, as assessed using the Wechsler Abbreviated Scale of Intelligence, 2nd ed. (WASI-II; 10/3/25 8:25:00 a.m.). All participants were verbally screened for known genetic or medical conditions potentially associated with secondary psychosis or ASD and excluded based on known conditions. Additional exclusionary criteria for all groups included contraindications for MRI scans (e.g., metal fragment injury, metal implants, metal orthodontic braces, or nonremovable retainers) to comply with scanner safety standards. Written informed assent was obtained from all participants under the age of 18, along with consent from parents/caregivers. Additionally, participants aged 18 years and older provided written consent, all using procedures approved by the Institutional Review Board at UCLA.

For the current study, we recruited a total of 77 participants. Prior to MRI scanning, all participants completed an MRI mock scanning session, using the MoTrak Head Motion Tracking System (PST-100722), which simulates a real MRI scan, to aid in reducing overall motion. Two participants were excluded from our sample due to either excessive head motion or technical artifact (see Supporting Information for additional details). Medication information for all groups can be found in Table 1.

### 2.2 | Rs-fcMRI Data Acquisition

Scans were acquired on a Siemens 3-Tesla Prisma scanner at UCLA using a 64-channel head coil. During the 8-min rs-fcMRI scan, subjects were told to relax and look at a white fixation cross in the center of a black background, presented through an LCD bold screen projector (TR = 720 ms, TE = 37.00 ms, field of view [FOV] = 208 mm, 72 slices, slice thickness = 2.0 mm, in plane–voxel size = 2.0 × 2.0 × 2.0 mm). For registration purposes, we additionally collected a high-resolution structural magnetization prepared–rapid acquisition gradient echo (MP-RAGE) scan for spatial registration of the rs-fcMRI scan to ensure identical distortion characteristics in a common space (TR = 2400 ms, TE = 2.22 ms, FOV = 256 mm, 208 slices, slice thickness = 0.8 mm, in plane-voxel size of 0.8 × 0.8 × 0.8 mm).

### 2.3 | Rs-fcMRI Data Processing

Rs-fcMRI data underwent standard preprocessing using FMRIB’s Software Library (FSL: Smith et al. 2004) and Analysis of Functional NeuroImages (AFNI; Cox 1996) including skull-stripping, slice-time correction, co-registration with the structural scan, motion correction, and regression of motion artifacts and white matter and CSF signals. See Supporting Information for detailed preprocessing steps.

### 2.4 | Rs-fcMRI Data Analysis

Our analysis followed a similar methodological approach to previous studies of seed-based whole brain functional connectivity of the DMN (Zhang and Raichle 2010; Funakoshi et al. 2016; Thomas et al. 2023), CEN (Dajani and Uddin 2016; Kaiser et al. 2015), and SN (Palaniyappan et al. 2013; Chen et al. 2016). Specifically, average residual time series were extracted from 5 mm radius seeds derived from prior studies (Table 2). The seed location for the DMN was the precuneus (MNI coordinates: −6, −52, 43; Fox et al. 2005), the right orbital fronto-insula (rOFI) for the SN (MNI coordinates: 38, 26, −10; Seeley et al. 2007), and the right dorsolateral prefrontal cortex (DLPFC) for the CEN (MNI coordinates: 44, 36, 20; Seeley et al. 2007). The time series extracted from each seed was subsequently correlated with every voxel in the brain to generate connectivity maps for each participant. The resulting connectivity map for each network was then converted into a *z*′-statistic map using Fisher’s *r*-to-*z* transform.

### 2.5 | Rs-fcMRI Between-Network Connectivity

All whole-brain group-level contrasts were conducted in AFNI, with variance estimated separately for each group. We were interested in examining the between-group whole-brain functional connectivity of the DMN, CEN, and SN. For each of the three networks, paired *t*-tests were conducted between each group to examine between-group differences in whole-brain connectivity. All statistical maps were adjusted for multiple comparisons to a corrected *p* < 0.001 using AFNI’s 3dClust-Sim. A general linear model was used to test if each cluster that exhibited a significant effect of group was over- or under-connected compared to TD controls. Connectivity *z*′-statistics were extracted from all significant clusters in AFNI and then correlated with behavioral measures with the Statistical Package for the Social Sciences (SPSS) Statistics software version 28.0 (IBM Corporation, Armonk, NY, USA). Additionally, in our rs-fcMRI analysis, we implemented cluster-level thresholding using Gaussian random field (GRF) theory to correct for multiple voxelwise comparisons. Clusters were considered significant at a family-wise error (FWE)-corrected threshold of *p* < 0.05, with an initial cluster-forming threshold of voxelwise *p* < 0.001.

### 2.6 | Behavioral Measures

To assess social cognition, all participants were administered The Awareness of Social Inference Test—Part 3 (TASIT; McDonald et al. 2006; see Supporting Information for detailed description of behavioral measures). A TASIT total raw score was calculated for each participant based on their responses to four questions regarding brief videos of naturalistic conversations between two actors across 16 vignettes, assessing participants’ understanding of social cues and ToM skills. To assess face emotion processing abilities, all participants were administered the WebCNP Emotion Recognition task (KER-40; Gur et al. 2010), in which they were instructed to examine a series of faces and identify the character’s expressed emotions. Participants were shown 40 color photographs of a static face expressing four basic emotions (anger, fear, happiness, and sadness) and neutral expressions. The task was presented in a randomized order, and the emotions shown ranged from low to high intensity. Performance was calculated as raw scores for accuracy and response time, which were first converted to *z*-scores and then subsequently converted to *T*-scores. Accuracy *T*-scores ranged from 0 to 70, with a higher score indicating better emotion recognition skills (Gur et al. 2010; Moore et al. 2015).

### 2.7 | Statistical Analysis

Statistical analyses were conducted using SPSS. First, we compared demographic variables between groups using independent sample *t*-tests for continuous variables such as age and socioeconomic status (SES) indexed as mean parental education years, and chi-square tests for categorical variables such as sex and race/ethnicity. Participants significantly differed on sex and ethnicity (Table 1); i.e., there were proportionally fewer female participants in the ASD group relative to the other groups, reflecting expected prevalence rates (Salari et al. 2022). While sex and ethnicity were not tested as independent predictors of behavioral or connectivity outcomes, these variables were included as covariates in all subsequent between-group analyses to account for potential confounding effects within the context of known diagnostic base rate differences (e.g., higher male prevalence in ASD). Additionally, the ASD and TD group had significantly more participants who identified as White, while the EOP group had significantly more participants who identified as being of Latinx origin compared to all other groups. Hence, sex and ethnicity (Hispanic/Not Hispanic) were included as covariates in all subsequent between-group analyses. Second, one-way analysis of covariances (ANCOVAs), were performed to examine between-group differences in total TASIT score and Emotion Recognition task accuracy scores (Table 3). Following this, pairwise comparisons were conducted to elucidate directionality of group differences for each significant ANCOVA. To examine how distinct rs-fcMRI connectivity patterns in each group were associated with impaired social cognition or emotion recognition, we then correlated the strength of functional connectivity (*z*′) extracted from the DMN, CEN, and SN whole-brain functional connectivity clusters for each group with behavioral measures, more specifically TASIT total scores, and KER-40 accuracy scores, covarying for sex and ethnicity. To minimize Type I error, we applied Bonferroni correction across all ANCOVAs and correlational analyses, adjusting *p*-values based on the total number of comparisons.

## 3 | Results

### 3.1 | Social Cognition Performance

Results from a one-way ANCOVA controlling for sex and ethnicity (Figure 1) revealed significant group differences. Pairwise differences showed that the total TASIT score was significantly lower in the ASD group compared to the TD [*F*(2, 73) = 3.78, *p* = 0.03]. Additionally, results from this one-way ANCOVA revealed that there were no significant group differences for total TASIT score [*F*(2, 73) = 1.00, *p* = 0.37] between ASD and EOP youth. Results from the second one-way ANCOVA revealed that there were no significant group differences for KER-40 task accuracy score [*F*(2, 56) = 0.11, *p* = 0.89].

### 3.2 | Triple Network Connectivity Within Each Group

To inspect the average connectivity of all three networks across all groups, we created masks from the within-group whole-brain connectivity patterns for TD, ASD, and EOP groups separately (see Supporting Information for details), thresholded at *p* < 0.001. This mask was formed by combining individual DMN, CEN, and SN resting state network maps for each group. Initially, we generated the individual network maps, and then we combined the three networks and applied a similar thresholding to the combined map. Results showed overall greater DMN connectivity in the precuneus hub in EOP youth compared to ASD and TD youth. ASD youth demonstrated overall greater CEN connectivity in frontal brain regions compared to parietal regions observed in the EOP and TD youth. Both ASD and EOP youth showed overall SN underconnectivity compared to TD youth (Figure 2).

### 3.3 | Between-Group Resting-State Network Maps

Significant between group differences were observed in all three networks (Figures 3A and S4A–S6C). Specifically, in the DMN, ASD participants exhibited precuneus hub overconnectivity with the bilateral fusiform gyrus and right middle occipital gyrus, but underconnectivity with the precuneus and left postcentral gyrus, compared to TD controls (*p* < 0.001; Figure S4A). EOP participants exhibited precuneus hub overconnectivity with the left middle frontal gyrus, left superior frontal gyrus, and left inferior occipital gyrus compared to TD controls (*p* < 0.001; Figure S4B). Compared to EOP participants, ASD participants also exhibited precuneus hub overconnectivity with the left frontal gyrus, but underconnectivity with the left middle frontal gyrus, left inferior frontal gyrus, and right hippocampus (*p* < 0.001; Figure S4C).

In the CEN, ASD participants exhibited DLPFC hub overconnectivity with the right inferior frontal gyrus and right caudate nucleus compared to TD controls (*p* < 0.001; Figures 3B and S5A). EOP participants exhibited DLPFC hub overconnectivity with the right postcentral gyrus and right Heschl’s gyrus compared to TD controls (*p* < 0.001; Figure S5B). ASD participants also exhibited DLPFC hub overconnectivity with the right superior frontal gyrus, right postcentral gyrus, left middle frontal gyrus, and right supplementary motor area compared to EOP participants (*p* < 0.001; Figure S5C).

In the SN, ASD participants exhibited rOFI hub underconnectivity with the left middle occipital gyrus, left inferior frontal gyrus, left postcentral gyrus, and left angular gyrus compared to TD controls (*p* < 0.001; Figures 3C and S6A). Between-group whole brain functional connectivity of the SN maps did not survive cluster correction for EOP participants compared to TD controls (*p* < 0.001; Figure S6B). ASD participants also exhibited rOFI hub underconnectivity with the left middle temporal gyrus, left middle occipital gyrus, and left precentral gyrus compared to EOP participants (*p* < 0.001; Figure S6C).

### 3.4 | Relationship Between Functional Connectivity and Behavioral Measures

In the EOP group, greater DMN positive connectivity was associated with poorer performance on TASIT total raw scores (*r* = −0.72, *p* = 0.002; Figure 4B), but not in the ASD and TD control groups. There were no significant associations between DMN connectivity and the KER-40 task accuracy *T*-scores observed in all three groups. In the ASD group, greater SN negative connectivity was associated with poorer performance on TASIT total raw scores (*r* = 0.46, *p* = 0.04; Figure 4A), but not in the EOP or TD control groups. There were no significant associations between SN connectivity and KER-40 task accuracy performance observed in all three groups. Lastly, there were also no significant associations between CEN connectivity and TASIT total raw scores or KER-40 accuracy *T*-scores observed in all three groups.

## 4 | Discussion

Here we used whole-brain rs-fcMRI to examine resting-state networks based on the triple-network model (DMN, CEN, and SN) in adolescents with ASD and EOP, relative to TD controls, as well as the relationship of network connectivity to social cognition and emotion recognition abilities. While social cognition deficits in ASD and psychosis have been well established (Baron-Cohen 2000; Couture et al. 2010; Eack et al. 2013; Penn et al. 2008; Pinkham et al. 2008), our results further illustrate that these deficits may be associated with atypical functional brain connectivity. To the best of our knowledge, this is the first study to directly examine functional connectivity of the triple-network in individuals with ASD, relative to EOP, and TD controls, in the crucial stage of brain development during adolescence. We found that the two clinical groups showed distinct relationships between functional connectivity of these networks and social cognition task performance.

Overall, the interplay of the DMN, CEN, and SN is essential for cognitive flexibility, which is the ability to adapt thinking and behavior in response to changing demands of social interactions (Uddin 2015; Kupis et al. 2020). These three networks collectively facilitate shifts between internally focused thought (DMN), external-directed cognitive control (CEN), and salient stimuli to mediate these transitions (SN; Schimmelpfennig et al. 2023). Disruptions within and between these core networks are thought to underlie diverse psychiatric conditions, including ASD and psychosis, which share overlapping social–cognitive deficits yet differ in their developmental trajectories and neurobiological substrates. Our results indicate some shared as well as distinct findings across ASD, EOP, and TD groups, specifically in our combined masks of DMN, CEN, and SN (Figure 2). Our functional connectivity results revealed that youth with ASD exhibited a pattern of mixed over- and under-connectivity within the DMN, overconnectivity in the CEN, and underconnectivity in the SN, compared to TD controls. In contrast, youth with EOP exhibited overconnectivity in the DMN and CEN, compared to TD controls.

In ASD youth, our findings of mixed DMN over- and underconnectivity relative to TD controls is consistent with prior studies (Assaf et al. 2010; Weng et al. 2010; Lynch et al. 2013; Redcay et al. 2013). Specifically, ASD participants showed DMN overconnectivity with regions involved in visual processing such as face processing and object recognition. ASD youth also showed DMN underconnectivity with the precuneus, left postcentral gyrus, and frontal gyrus compared to both TD groups. In contrast, our findings of CEN overconnectivity in ASD youth compared to TD controls, is inconsistent with prior studies (Sherman et al. 2014; Bi et al. 2018; Mouga et al. 2022), that have found mixed CEN over- and underconnectivity patterns. Specifically, ASD youth exhibited CEN overconnectivity with the inferior frontal gyrus, postcentral gyrus, middle and superior frontal regions, areas involved in attention, working memory, semantic processing, and sensory integration, compared to TD controls. On the other hand, SN underconnectivity in ASD youth was observed in regions involved in language, auditory processing, and motor planning and was associated with poorer performance on a social cognition task. This suggests SN-mediated difficulties in regulating communication, sensory integration, and visual–social processing in ASD.

In the EOP group, participants demonstrated DMN overconnectivity in regions associated with working memory and visual processing, consistent with previous findings (Guo et al. 2014; Cui et al. 2017; Sasabayashi et al. 2023). Importantly, this observed DMN overconnectivity was associated with poorer performance on a social inference task, suggesting that over-recruitment of DMN hubs may reflect potential compensatory social information processing in this group. Similarly, CEN overconnectivity in the EOP group encompassed regions associated with executive function and working memory, consistent with previous research linking CEN alterations to cognitive disorganization in psychosis (Achim et al. 2021; Anhøj et al. 2018; Gur et al. 2014; Karcher et al. 2022; Kong et al. 2013; Manoliu et al. 2014). By contrast, SN differences in EOP youth did not survive cluster correction. This divergence from prior studies reporting mixed SN findings in psychosis (Du et al. 2018; Ohta et al. 2018) may reflect sample size differences, age-related variability, or symptom-specific effects.

Direct comparisons between ASD and EOP further highlighted distinct triple-network connectivity disruptions in each group. First, while both clinical groups showed DMN-related alterations, the patterns differed with ASD youth showing mixed over- and under-DMN connectivity even compared to EOP youth (who only demonstrated DMN overconnectivity compared to TD controls). However, in the EOP group, greater DMN connectivity was associated with poorer social cognition performance, suggesting maladaptive engagement of self-referential processes during social inference making. DMN overconnectivity in EOP may actually hinder rather than support social cognitive abilities. These findings suggest that both groups experience disruptions in self-referential processing and theory of mind, core functions subserved by the DMN (Menon 2011; Uddin et al. 2008; Lombardo et al. 2010), though these disruptions may reflect divergent neurodevelopmental pathways (Palaniyappan and Liddle 2012; Sha et al. 2022). Second, for the CEN, ASD youth demonstrated overconnectivity compared to EOP youth; however, while CEN disruptions in EOP may relate to overall cognitive disorganization (Dey et al. 2021), CEN recruitment in ASD may reflect engagement in support of social cognitive demands (Bi et al. 2018; Mouga et al. 2022). Though we did not include executive functioning measures in this study, future research could clarify how these distinct CEN connectivity patterns relate to overt cognitive control behaviors across groups. Lastly, SN underconnectivity was specific to ASD compared to EOP youth, which was further associated with poorer social cognition, consistent with prior literature that salience detection impairments disrupt social cue processing (Margolis et al. 2019). Taken together, these results show that while both clinical groups may share overlapping impairments in social cognition, the underlying neural pathways differ such that SN underconnectivity may have a more direct, developmentally rooted role in ASD, disrupting early salience detection and development of reciprocal social interaction skills and social motivation (Morrison et al. 2017), whereas EOP youth show DMN overconnectivity hindering social expression and relatedly inference making (Morrison et al. 2017; Pinkham et al. 2020).

Our connectivity findings differ from those observed in prior studies examining the roles of the triple-network in ASD and individuals with psychosis, wherein functional overconnectivity in the DMN was found in children with ASD (Menon 2018; Li et al. 2022) as well as in adults with psychosis (Krishnadas et al. 2014) compared to TD controls. For instance, Menon (2018) found overconnectivity in the DMN among children with ASD compared to adolescents with ASD, reflecting potential age-related changes in network connectivity. Yue et al. (2022) identified specific network interactions in adults with ASD, including increased connectivity within the CEN and SN, as well as increased connectivity between the DMN and SN, relative to controls. Krishnadas et al. (2014) reported overconnectivity within the DMN, CEN, and SN in adults with paranoid schizophrenia, compared to healthy controls. Supekar et al. (2019) found dysregulation within and between the DMN, CEN, and SN in psychosis, noting overconnectivity within individual networks in the DMN and SN, and reduced connectivity within network connectivity in the CEN, in addition to associations between these networks, relative to healthy controls. It is likely that some of the differences in findings could be attributed to age-related differences in study samples as well as differences in methodological techniques used (such as connectivity analysis restricted to known voxels within each network examined in some prior studies, versus whole-brain connectivity of hubs of individual networks in our study), use of different comparison samples in our study, as well as differential inclusion criteria within the psychosis sample across studies.

Despite these differences, the overall trend of impacted connectivity in the triple-network model aligns well with our findings and, cumulatively, these findings suggest that these distinct connectivity patterns within the DMN, CEN, and SN may be relevant to overt social behaviors and face-emotion processing difficulties observed in each of these neuropsychiatric conditions. Our findings emphasize the further need to explore different domains of social cognition, such as social inference making and emotional regulation in well-matched groups of individuals with ASD and EOP. A network-based framework provides a meaningful lens for better understanding both shared vulnerabilities and disorder-specific pathways across neurodevelopmental conditions. Moreover, by identifying where ASD and EOP diverge in their network-behavior associations, our results highlight the importance of tailoring interventions. Finally, while ASD and EOP present with distinct developmental trajectories, both involve dysconnectivity in networks that regulate the balance between self-focused thought, external cognitive control, and salience detection. By directly linking triple-network connectivity to performance on validated behavioral measures, these results demonstrate neural differences and map onto real-world social difficulties. Future research should incorporate longitudinal studies to assess social cognitive differences across developmental stages in individuals with ASD and EOP, given the dynamic brain changes occurring across this neurodevelopmental period.

### 4.1 | Limitations

The main limitation of this study is that the sample size in our study is relatively small, given the challenges of recruiting well-matched clinical populations for neuroimaging studies, especially those with lower prevalence rates such as EOP. Further, there was a limited range of available behavioral measures as part of this study. It is likely that other social cognition measures (and related executive functioning measures) could better account for the relationship to brain regions involved in the triple-network model. Furthermore, while we ethically chose not to alter medication regimens for participants, this resulted in a sample of mixed medication history across groups (most ASD participants were not on current medication, and several EOP participants had recently begun medication) that may influence the findings. Further research is needed to examine other aspects of social cognition in the triple-network model as well as inclusion of more comprehensive behavioral measures of social cognition and related higher-order cognitive processes. Despite these limitations, our results provide new information regarding dysconnectivity patterns within the triple-network across neuropsychiatric groups.

## 5 | Conclusion

In conclusion, our findings demonstrate that youth with ASD exhibited mixed over- and underconnectivity of the DMN, overconnectivity of the CEN, and underconnectivity of the SN compared to TD controls. Similarly, youth with EOP exhibited DMN overconnectivity and CEN overconnectivity compared to TD controls, while functional connectivity of the SN did not survive cluster corrections. Notably, our findings also demonstrate that youth with ASD exhibited mixed over- and under-connectivity of the DMN, overconnectivity of the CEN, and underconnectivity of the SN compared to EOP youth. Further, our results suggest that while social cognition and face–emotion processing abilities may be associated with DMN and SN functional connectivity across these groups, they differ in their brain–behavior association patterns. These results contribute to the understanding of the social vulnerabilities in these neuropsychiatric conditions. As both ASD and EOP share several overlapping genotypic and phenotypic features, the direct comparison of these two clinical populations is valuable for understanding distinct and overlapping functional connectivity patterns, and contributes to our understanding of differential social cognition abilities across these clinical populations. Our study helps in elucidating disrupted neurodevelopmental mechanisms that underlie the phenotypic spectrum of both neuropsychiatric conditions and how it relates to social cognition deficits. Future studies can further advance our knowledge of these mechanisms by incorporating larger sample sizes, diverse behavioral measures, and longitudinal designs to better capture the developmental trajectories and implications for youth with ASD and EOP.

## Supplementary Material

Supplementary Materials

Additional supporting information can be found online in the Supporting Information section. Data S1: aur70163-sup-0001-Supplementary_Material.docx.

## Figures and Tables

**FIGURE 1 |:**
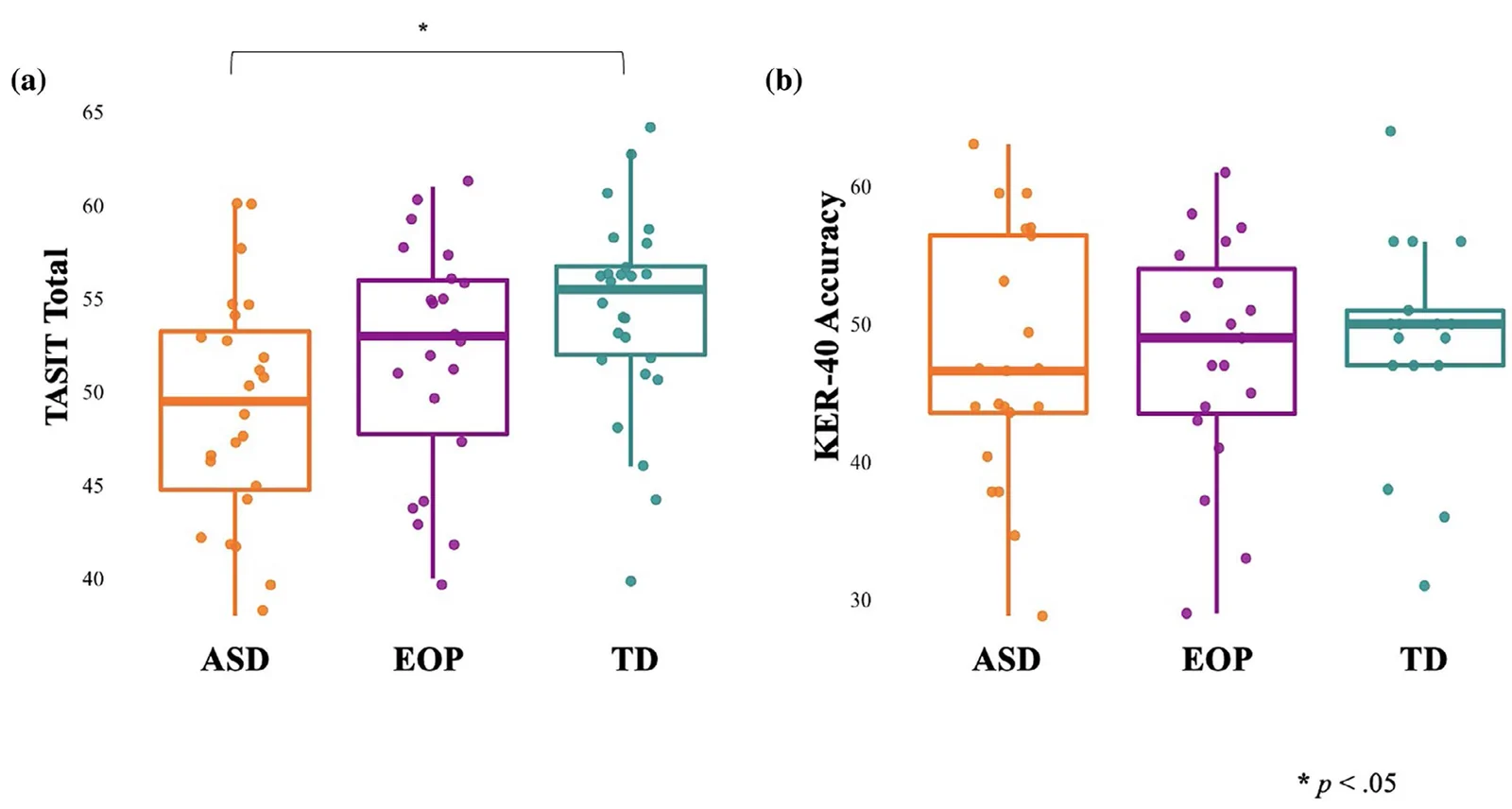
Boxplots showing group differences in TASIT total, KER-40 accuracy, and KER-40 response time scores. Lower and upper box boundaries indicate 25th and 75th percentiles, respectively, bold line inside box represents median, whiskers denote minimum to maximum range, and significant pairwise differences between groups are indicated by asterisks (*p < 0.05). (A) Group differences in TASIT total (ASD, EOP, TD). (B) Group differences in KER-40 accuracy (ASD, EOP, TD).

**FIGURE 2 |:**
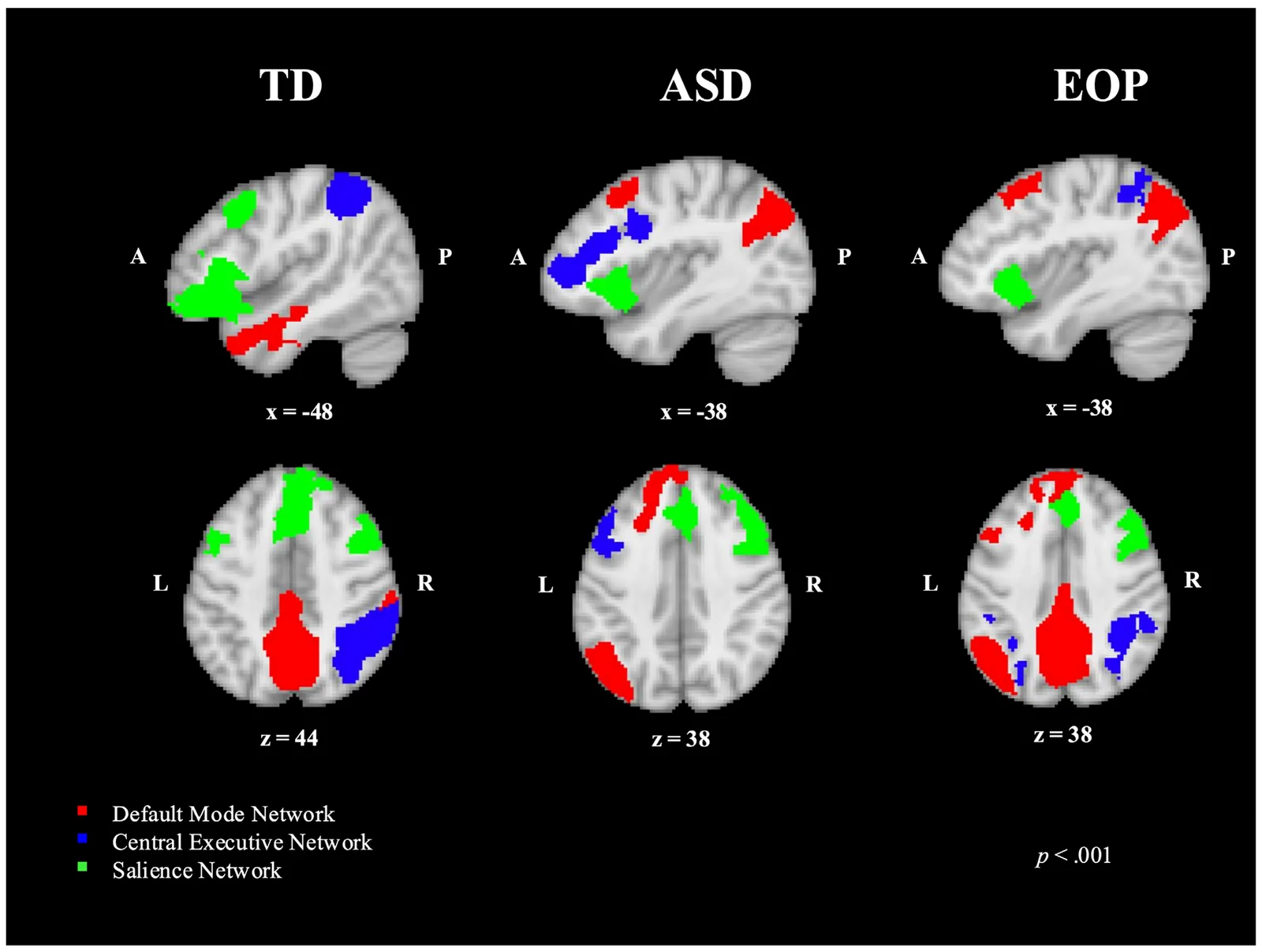
Prethresholded mask combining individual DMN, CEN, and SN resting state network maps for each group: TD, ASD, and EOP. DMN maps are shown in red, CEN maps are shown in blue, and SN maps are shown in green on a MNI152 2 mm standard image.

**FIGURE 3 |:**
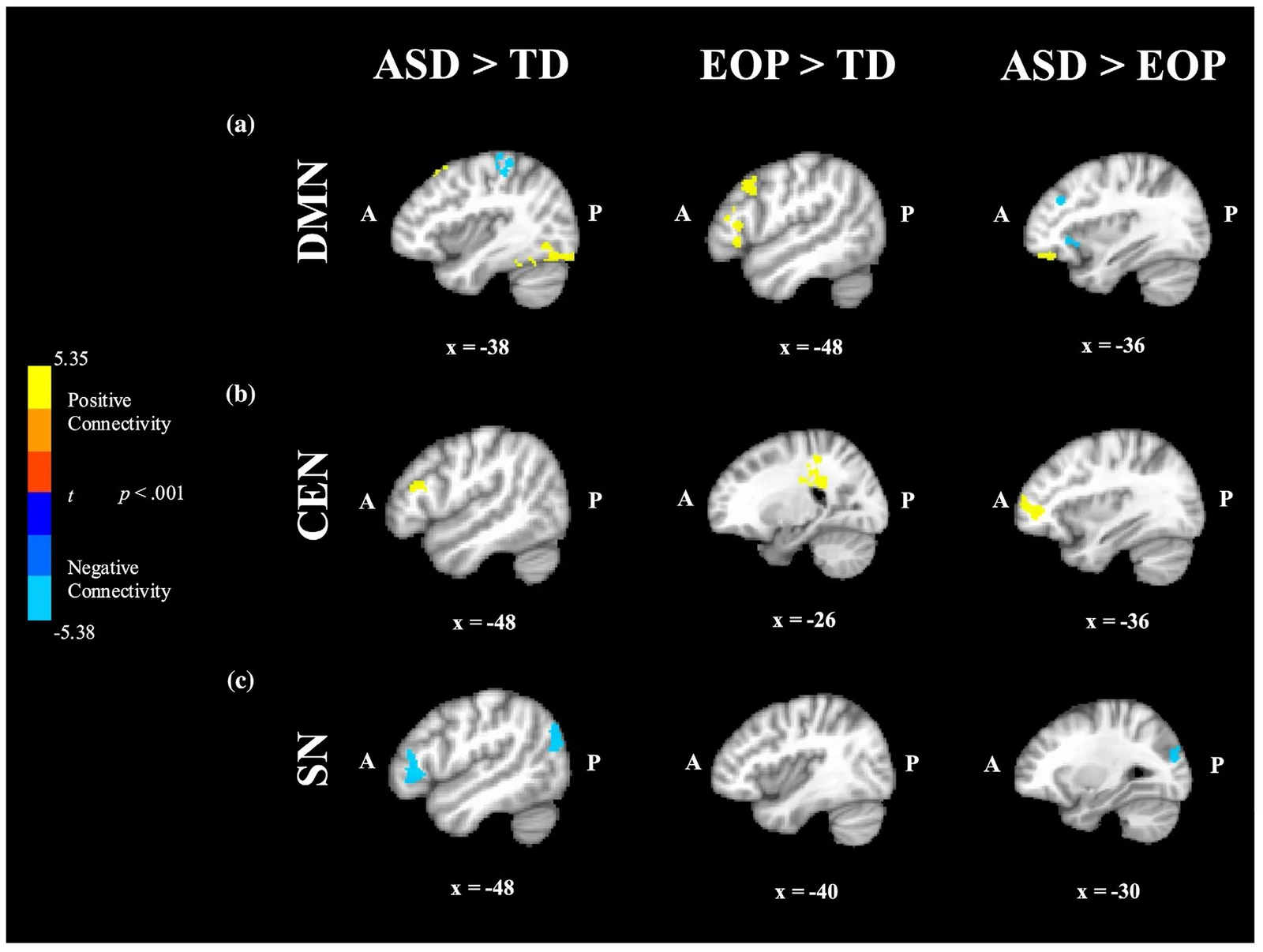
(A) Between-group seed-based whole brain resting state functional connectivity of the DMN. ASD participants exhibited mixed over-and-under-connectivity of DMN compared to TD controls (*p* < 0.001). EOP participants exhibited DMN overconnectivity compared to TD controls (*p* < 0.001). ASD participants also exhibited mixed over-and-under-connectivity of DMN compared to EOP participants (*p* < 0.001). (B) Between-group seed-based whole brain functional connectivity of the CEN. ASD participants exhibited overconnectivity of CEN compared to TD controls (*p* < 0.001). EOP participants exhibited overconnectivity of CEN compared to TD controls (*p* < 0.001). ASD participants also exhibited CEN overconnectivity compared to EOP participants (*p* < 0.001). (C) Between-group seed-based whole brain resting state functional connectivity of the SN. ASD participants exhibited SN underconnectivity compared to TD controls (*p* < 0.001). Between-group seed-based whole brain resting state functional connectivity of the SN did not survive cluster corrections for EOP participants compared to TD controls (*p* < 0.001). ASD participants also exhibited SN underconnectivity compared to EOP participants (*p* < 0.001).

**FIGURE 4 |:**
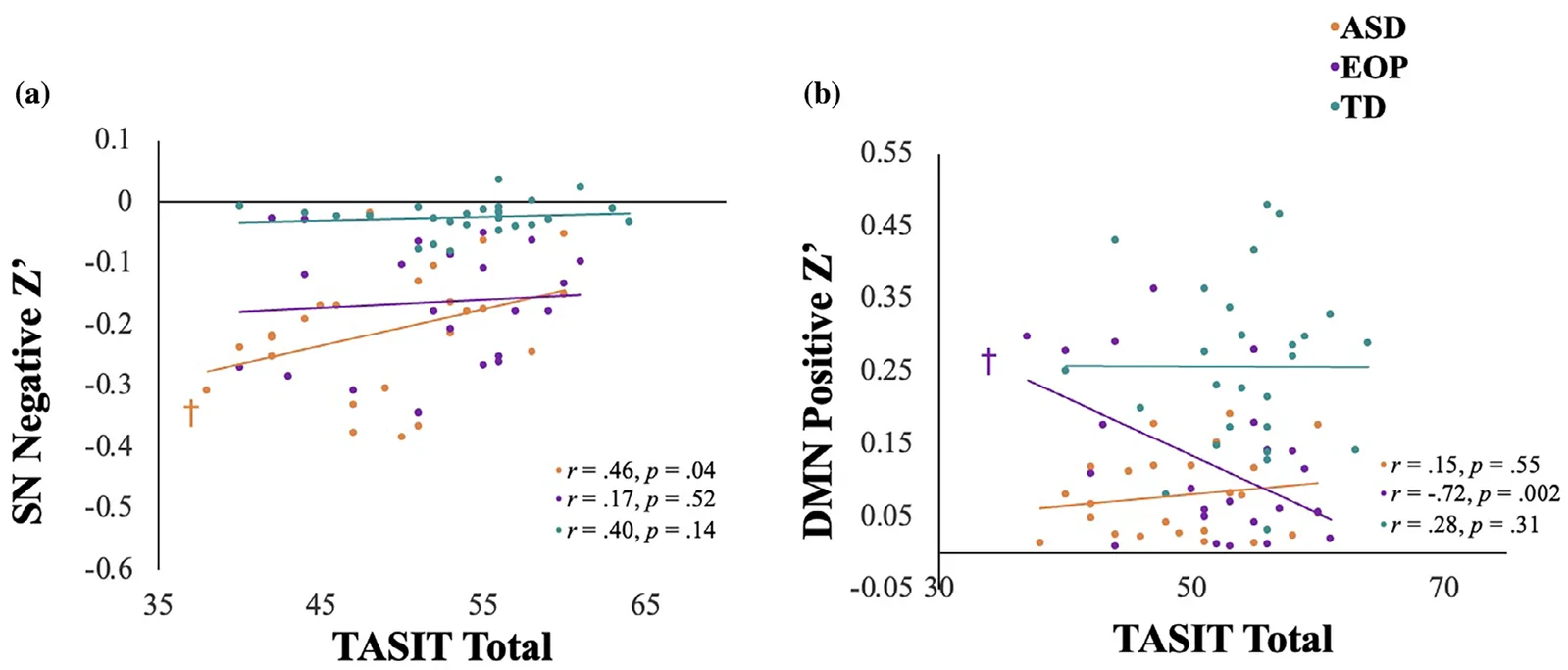
(A) Correlation between rs-fcMRI SN negative *z*′ scores and TASIT total for ASD, EOP, and TD. (B) Correlation between rs-fcMRI DMN positive *z*′ scores and TASIT total for ASD, EOP, and TD.

**TABLE 1 |:** Demographic data for participant groups.

	ASD (*N* = 24), mean (SD)	EOP (*N* = 25), mean (SD)	TD (*N* = 26), mean (SD)	*p*
Mean age, in years (±SD)	15.89 (±2.12)	16.42 (±2.13)	16.86 (±2.69)	0.35
Percent female (*N*)	13.0% (3F)**	52.0% (13F)**	58.0% (15F)**	0.002
FSIQ	110.67 (16.41)	105.28 (12.84)*	119.33 (12.78)*	0.01
Nonverbal IQ (scaled score)	113.61 (16.73)	104.05 (15.21)	111.80 (15.52)	0.11
Mean parental education, in years (±SD)	15 (2.66)	16 (2.13)	17 (2.61)	0.11
Ethnicity				0.03
White, Hispanic	33%	44%*	12%*	
White, non-Hispanic	67%	56%*	88%*	
Medication				0.001
SSRI	1	1	0	
Antipsychotic	1	2	0	
SSRI + antipsychotic	4*	15*	0	
Stimulant	0	1	0	
No medications	18	6*	26*	

Abbreviations: FSIQ, full scale IQ; SD, standard deviation; SSRI, selective serotonin reuptake inhibitor.

* *p* < 0.05.

** *p* < 0.01.

**TABLE 2 |:** MNI coordinates of seed-based resting-state functional connectivity.

Seed	Brain region	*X*	*Y*	*Z*
Default mode network	Left precuneus	−6	−52	43
Central executive network	Right dorsolateral prefrontal cortex	44	36	20
Salience network	Right orbital frontoinsula	38	26	−10

**TABLE 3 |:** Group means and standard deviations for TASIT total, emotion recognition task accuracy and speed, and *z*′ connectivity scores.

	ASD, mean (SD)	EOP, mean (SD)	TD, mean (SD)	*p*
TASIT total (*T*-score)	49.57 (6.11)*	51.57 (6.11)	54.24 (5.56)*	0.03
KER-40 accuracy (*T*-score)	47.50 (9.11)	47.00 (7.65)	49.44 (7.46)	0.68
KER-40 response time (*T*-score)	44.71 (9.78)	42.07 (8.54)	48.38 (10.30)	0.20
Average DMN *Z*′ positive connectivity	0.39 (0.07)***	0.43 (0.10)***	0.26 (0.12)***	< 0.001
Average SN *Z*′ negative connectivity	−0.02 (0.02)***	−0.14 (0.06)***	−0.02 (0.03)***	< 0.001
ADOS-2 average CSS	6.25 (1.57)	—	—	—
SCID-5 diagnosis (%)	—			
Schizophrenia		12%	0%	
Schizoaffective		28%	0%	
Schizophreniform		0%	0%	
Bipolar I w/psychosis		4%	0%	
Major depressive disorder w/psychosis		12%	0%	
Psychotic disorder not otherwise specified		44%	0%	

Abbreviations: ADOS-2, autism diagnostic observation schedule, 2nd edition; CSS, calibrated severity score; DMN, default mode network; KER-40, WebCNP Emotion Recognition Task; SCID-5, structured clinical interview for DSM-5; FSIQ, full scale IQ; SD, standard deviation; SN, salience network; TASIT, The Awareness of Social Inference Test—Part 3.

* *p* < 0.05.

*** *p* < 0.001.

## Data Availability

The original contributions presented in this study are included in this article/Supporting Information; further inquiries may be directed to the corresponding authors.
