# Supplementary material for: Triple-Network Functional Connectivity in Adolescents With Autism Spectrum Disorder Compared to Early-Onset Psychosis
Source: Autism Res. Author manuscript; Available in PMC 2026 Oct 12. (PMC13657677; doi:10.1002/aur.70163)
Supplement: Supplementary file 1 [file NIHMS2212745-supplement-Supplementary_Materials.pdf]

SUPPLEMENTARY MATERIAL

Materials and Methods

Participants

For the current study, we recruited a total of 77 participants. Prior to MRI scanning, all participants completed an MRI mock scanning session, using the MoTrak® Head Motion Tracking System (PST-100722), which simulates a real MRI scan, to aid in reducing overall motion. Two participants were excluded from our sample due to either excessive head motion or technical artifact. One ASD participant was excluded from our sample due to excessive head motion during MRI scanning (root mean square of the sum of displacement motion > 0.5 mm; See rs-fcMRI Data Processing section for details), and one EOP participant was excluded due to detection of a metal artifact during their scan.

Rs-fcMRI Data Processing

Rs-fcMRI data for each time point underwent standard preprocessing using FMRIB's Software Library (FSL: Smith *et al*., 2004) and Analysis of Functional NeuroImages (AFNI; Cox, 1996). This preprocessing step included skull stripping in AFNI, slice-time and head motion correction in FSL using the Motion Correction Linear Registration Tool (Jenkinson et al., 2002). Affine registration was conducted with 6 degrees of freedom for each subject's high-resolution matched coplanar anatomical image using FMRIB's Linear Image Registration Tool and Non-linear Image Registration Tool (FLIRT; FNIRT; Jenkinson *et al*., 2002; Jenkinson and Smith, 2001). For standardization to an anatomical framework, this was followed by affine registration with 12 degrees of freedom to the standard MNI template of 152 averaged brains (2mm isotropic resolution) using FLIRT. As part of quality control procedures, each registration was visually inspected. Images were spatially smoothed with a 6 mm full width at half maximum (FWHM) Gaussian kernel, using AFNI's 3dBlurToFWHM. The automatic Independent Component Analysis (ICA)-based Automatic Removal of Motion Artifacts (Pruim *et al*., 2015(a); Pruim *et al*., 2015(b)) was additionally used to remove potential motion confounds and regress out components labeled as either motion or noise. Images were spatially smoothed with a 6 mm full width at half maximum (FWHM) Gaussian kernel, using AFNI's 3dBlurToFWHM.

Multiple steps beyond conventional motion correction were taken during the preprocessing step to reduce the effect of head motion. Average head motion for each time point, defined as root mean square of the sum displacement (RMSD) of all six translational and rotational axes, was determined for each participant. Motion regressors, including 6 rigid-body motion parameters and their derivatives, were removed from the time series. Time points with excessive head motion with any instance of RMSD > 0.5mm, was censored including one time point immediately preceding and following motion. Any time point that did not belong to a series of at least 10 consecutive time points remaining after censoring was discarded and only participants with >80% time points remaining were included. Average signal was extracted at each timepoint from white matter and cerebrospinal fluid masks using AFNI's 3dmaskave. Further, to reduce noise and other confounds, data were bandpass filtered (0.01 Hz $< f <$ 0.1 Hz) using AFNI, to isolate frequencies at which intrinsic network-specific blood oxygen level-dependent correlations are known to predominate (Cordes *et al*., 2001). Nuisance regressors including mean white matter time series, mean cerebrospinal fluid time series, and temporal derivatives were removed (Power *et al*., 2014). Regressing out global signal from the data typically introduces negative correlations (Gotts *et al*., 2013; Liu *et al*., 2017; Murphy *et al*., 2009); therefore, global signal regression was not performed in our analyses.

Behavioral Measures

To assess social cognition, all participants were administered The Awareness of Social Inference Test - Part 3 (TASIT; McDonald *et al*., 2006). The TASIT has been widely validated across different clinical populations, including high-functioning adults with ASD *(Mathersul et al*., 2013), patients with schizophrenia (Kern, *et al*., 2009; Sparks *et al.,* 2010), and in individuals with traumatic brain injury (McDonald *et al*., 2003). The TASIT consists of 16-videotaped vignettes (each lasting between 15 to 60 seconds) of everyday social situations, assessing one's ability to draw inferences about the thoughts, intentions, beliefs, and feelings of others engaged in conversational exchanges involving white lies or sarcasm. After viewing each vignette, participants are asked to answer four types of yes or no questions addressing different aspects of communicative intentions of the characters. The first question asked what one character in the scene was doing (Do), the second question asked what the character was trying to say to the other person (Say), the third question asked what the main character was thinking (Think), and the fourth question asked what the main character was

feeling (Feel). A TASIT total score was calculated for each participant by adding their responses to each of the four questions per scene across all 16 vignettes, ranging from 0 to 64.

Rs-fcMRI Within-Network Connectivity

One sample *t*-tests were conducted in AFNI to examine the connectivity within each network of each group. We were primarily interested in seed-based whole brain functional connectivity of the DMN, CEN, and SN; thresholded at $p < 0.001$ within the ASD, EOP, and TD groups.

Results

Within-Group Resting-State Network Maps

For the DMN, TD controls exhibited precuneus whole-brain positive connectivity with the cingulate cortex, middle orbital gyrus, and right middle temporal gyrus ($p < .001$; Supplementary Figure 1A). ASD participants exhibited precuneus whole-brain positive connectivity with orbital gyrus, left angular gyrus, and left middle temporal gyrus; but negative connectivity with the right insula ($p < .001$; Supplementary Figure 1B). EOP participants exhibited positive whole-brain connectivity of the precuneus with the cingulate cortex, left middle frontal gyrus, and left angular gyrus; but negative connectivity with the right inferior frontal gyrus ($p < .001$; Supplementary Figure 1C). For the CEN, TD controls exhibited whole-brain DLPFC positive connectivity with the right inferior parietal lobule and left inferior frontal gyrus ($p < .001$; Supplementary Figure 2A). ASD participants exhibited whole-brain DLPFC positive connectivity with the right inferior parietal lobule and left inferior frontal gyrus ($p < .001$; Supplementary Figure 2B). EOP participants exhibited whole-brain DLPFC positive connectivity with the right inferior frontal gyrus and bilateral parietal lobule ($p < .001$; Supplementary Figure 2C). For the SN, TD controls exhibited whole-brain rOFI positive connectivity with the anterior cingulate cortex and left insula ($p < .001$; Supplementary Figure 3A). ASD participants exhibited whole-brain rOFI positive connectivity with the anterior cingulate cortex and left insula ($p < .001$; Supplementary Figure 3B). Lastly, EOP participants exhibited rOFI whole-brain positive connectivity with the left insula and anterior cingulate cortex ($p < .001$; Supplementary Figure 3C). Group means and standard deviations for z' connectivity scores can be found in Table 3. See supplementary figures 1A-3C for additional views.

Supplementary Figures

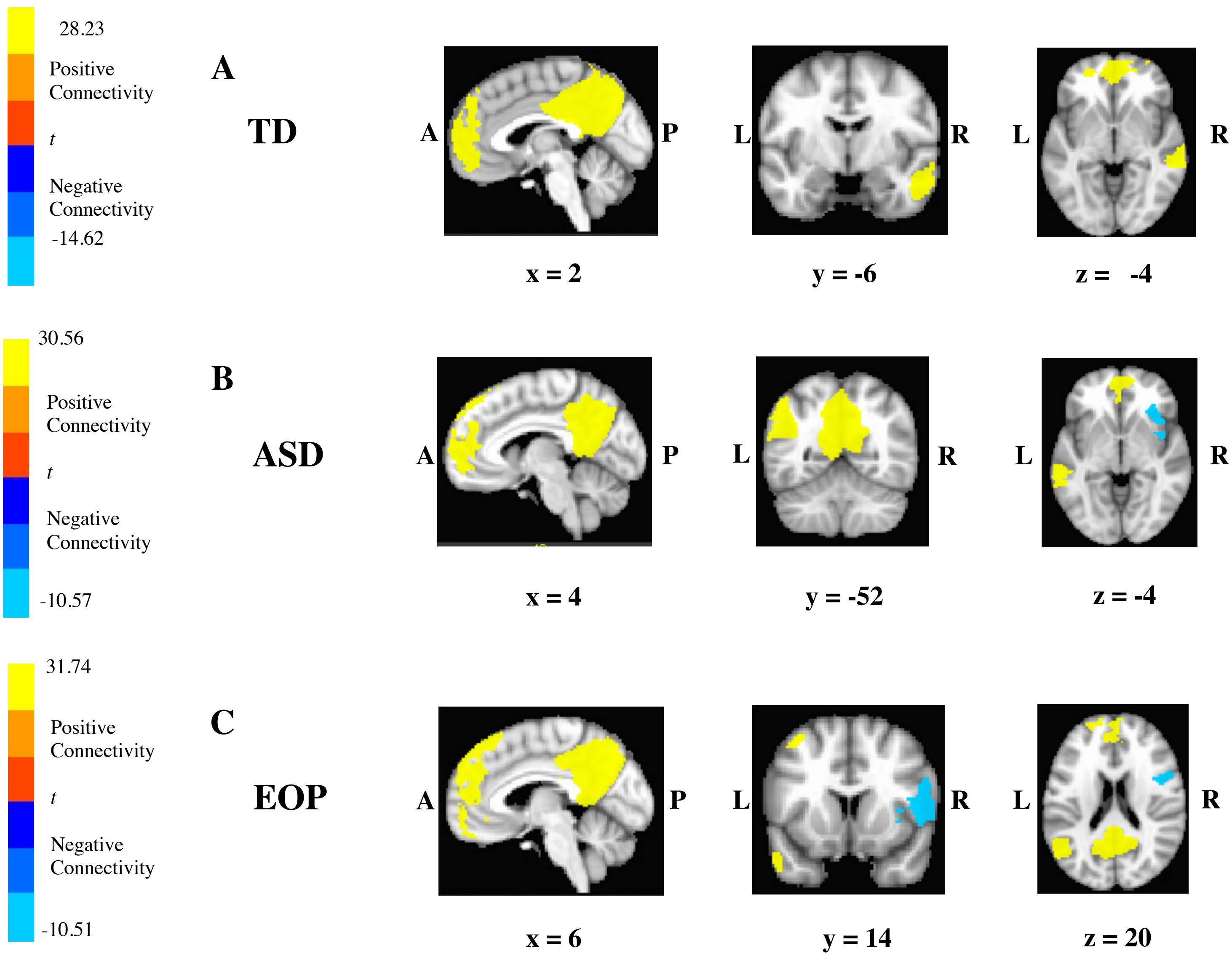

Supplementary Figure 1: Within-group seed-based whole brain resting state functional connectivity of the precuneus hub of the Default Mode Network, by group. TD controls exhibited precuneus positive connectivity with the posterior cingulate cortex, middle orbital gyrus, and right middle temporal gyrus ($p < .001$). ASD participants exhibited precuneus positive connectivity with orbital gyrus, left angular gyrus, and left middle temporal gyrus; but negative connectivity with the right insula ($p < .001$). EOP participants exhibited precuneus positive connectivity with the posterior cingulate cortex, left middle frontal gyrus, and left angular gyrus; but negative connectivity with the right inferior frontal gyrus ($p < .001$).

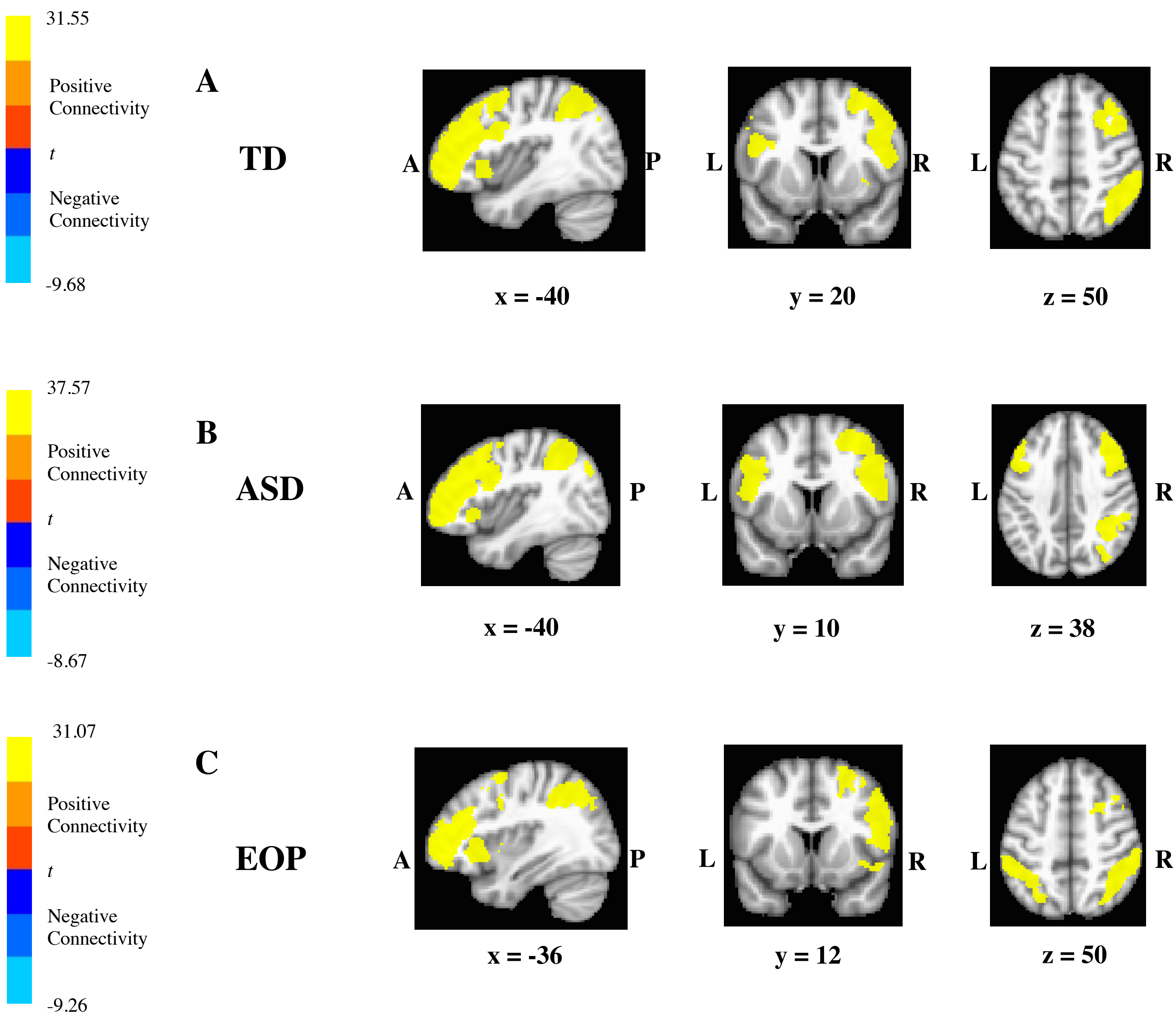

Supplementary Figure 2: Within-group seed-based whole brain functional connectivity of the right dorsolateral prefrontal cortex hub of the Central Executive Network, by group. TD controls exhibited DLPFC positive connectivity with the right inferior parietal lobule and left inferior frontal gyrus ($p < .001$). ASD participants exhibited DLPFC positive connectivity with the right inferior parietal lobule and left inferior frontal gyrus ($p < .001$). EOP participants exhibited DLPFC positive connectivity with the bilateral parietal lobules ($p < .001$).

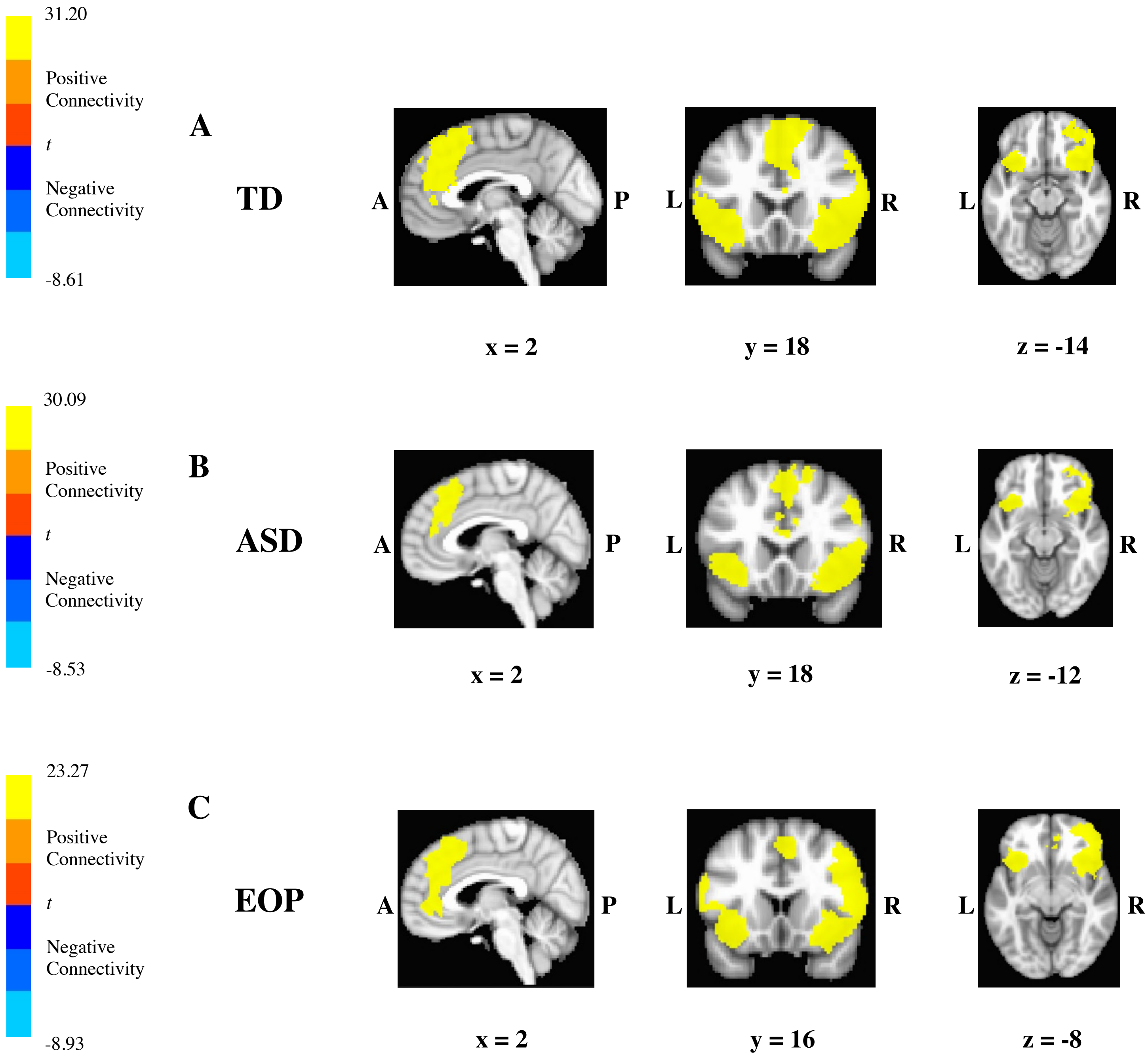

Supplementary Figure 3 Within-group seed-based whole brain functional connectivity of the right orbital frontoinsula hub of the Salience Network, within groups. TD controls exhibited right insula positive connectivity with the anterior cingulate cortex and left insula ($p < .001$). ASD participants exhibited right insula positive connectivity with the anterior cingulate cortex and left insula ($p < .001$). EOP participants exhibited right insula positive connectivity with the anterior cingulate cortex and left insula ($p < .001$).

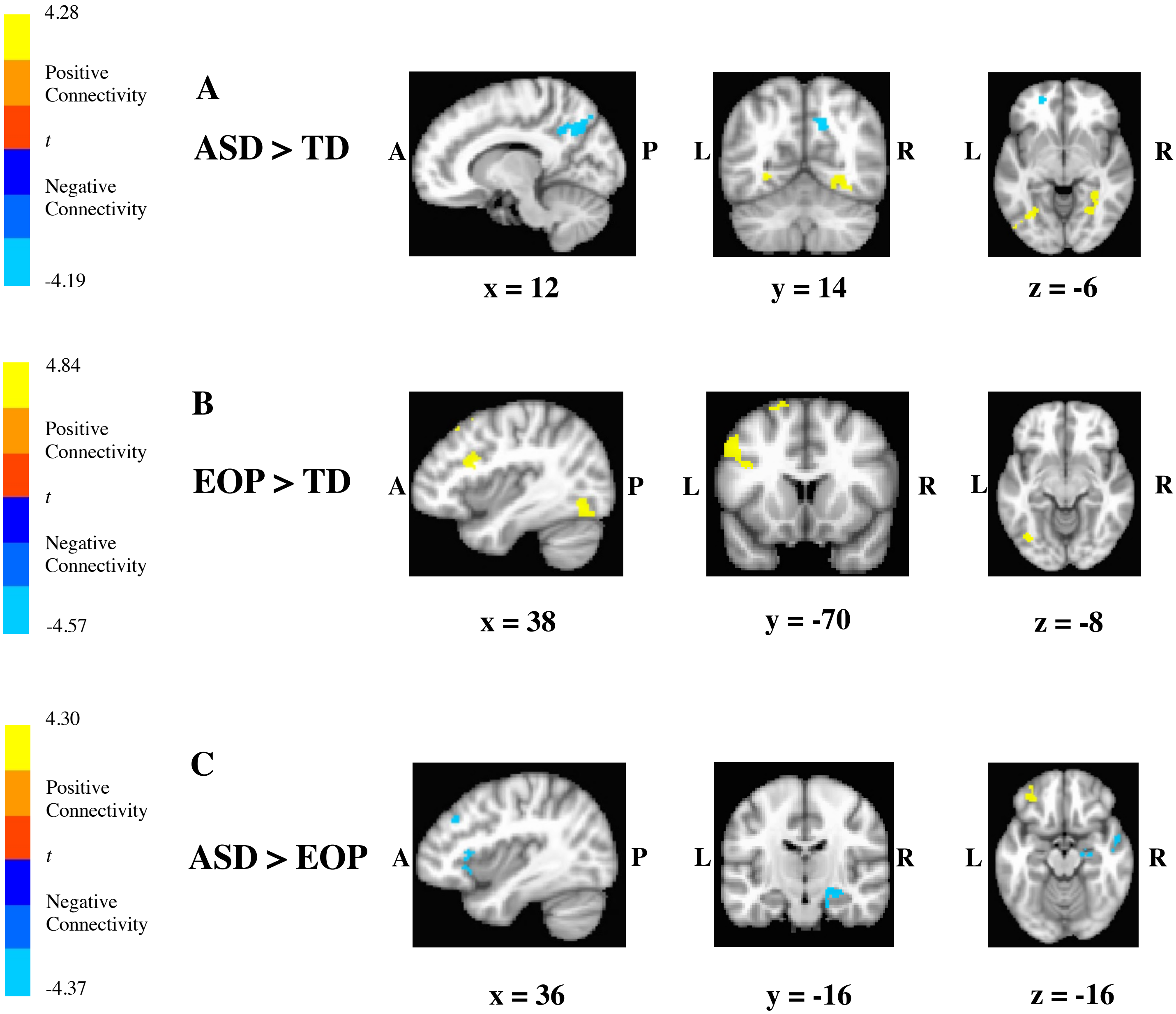

Supplementary Figure 4 Between-group seed-based whole brain resting state functional connectivity of the precuneus hub of the Default Mode Network. ASD participants exhibited overconnectivity with the bilateral fusiform gyrus and right middle occipital gyrus; but underconnectivity with the right precuneus and left postcentral gyrus compared to TD controls ($p < .001$). EOP participants exhibited overconnectivity with the left middle frontal gyrus, left inferior frontal gyrus, and left inferior occipital gyrus compared to TD controls ($p < .001$). ASD participants also exhibited overconnectivity with the left frontal gyrus; but underconnectivity with the left middle frontal gyrus, left inferior frontal gyrus, and right hippocampus compared to EOP participants ($p < .001$).

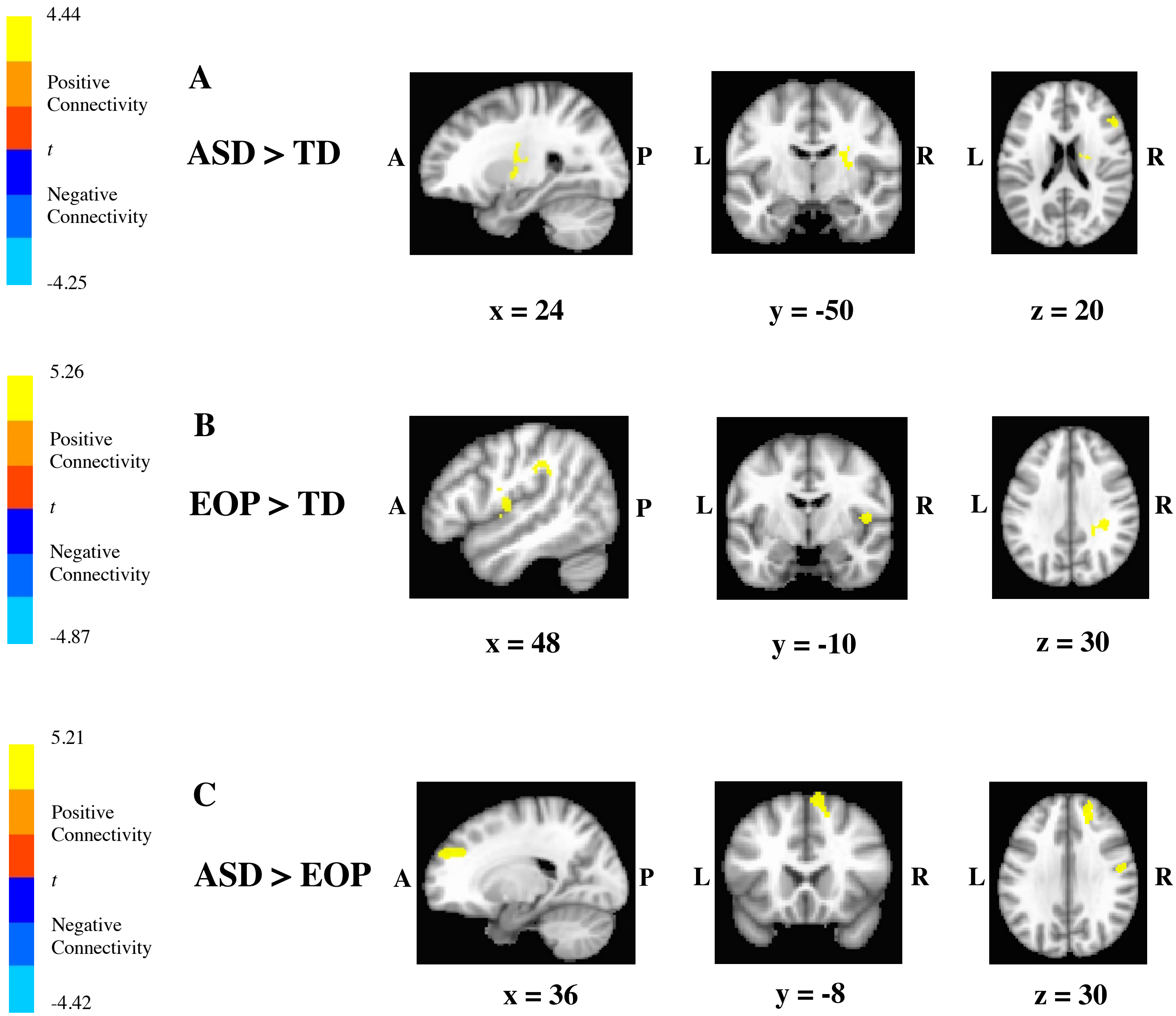

Supplementary Figure 5 Between-group seed-based whole brain functional connectivity of the dorsolateral prefrontal cortex hub of the Central Executive Network. ASD participants exhibited overconnectivity with the right inferior frontal gyrus and right caudate nucleus compared to TD controls ($p < .001$). EOP participants exhibited overconnectivity with the right postcentral gyrus and right Heschl's gyrus compared to TD controls ($p < .001$). ASD participants also exhibited overconnectivity with the right superior frontal gyrus, right postcentral gyrus, left middle frontal gyrus, and right supplementary motor area compared to EOP participants ($p < .001$).

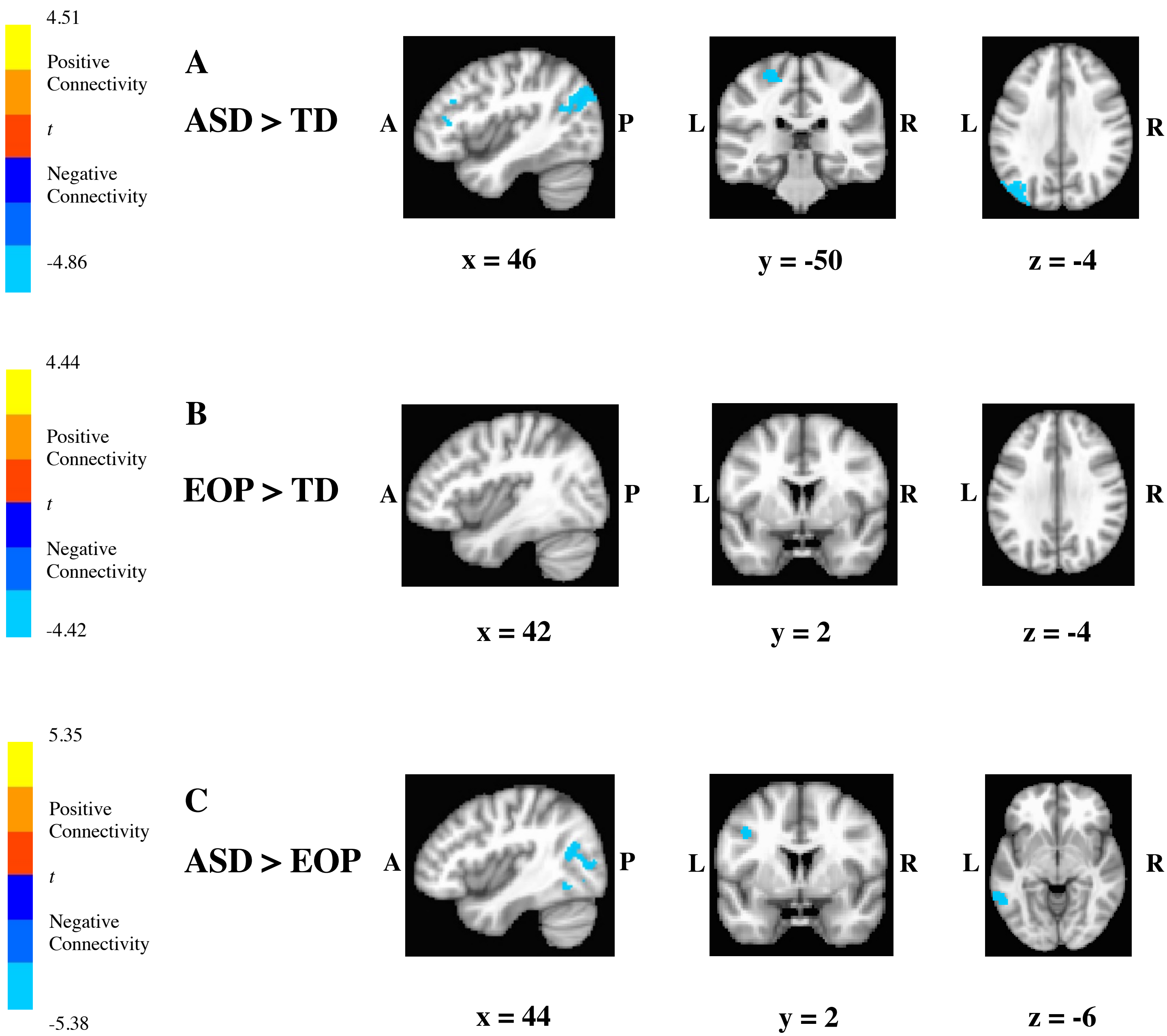

Supplementary Figure 6 Between-group seed-based whole brain functional connectivity of the right orbital frontoinsula hub of the Salience Network, ASD participants exhibited underconnectivity with the left middle occipital gyrus, left inferior frontal gyrus, left postcentral gyrus, and left angular gyrus compared to TD controls ($p < .001$). Between-group whole brain functional connectivity of the SN maps did not survive cluster correction for EOP participants compared to TD controls ($p < .001$). ASD participants also exhibited underconnectivity with the left middle temporal gyrus, left middle occipital gyrus, and left precentral gyrus compared to EOP participants ($p < .001$).
